# Disease modifying treatment of hereditary transthyretin amyloidosis with polyneuropathy in Germany – expert consensus of the German society of amyloid diseases (DGAK) and the German neurological society (DGN)

**DOI:** 10.1186/s42466-026-00521-4

**Published:** 2026-08-11

**Authors:** Markus Weiler, Maike F. Dohrn, Stefan Gingele, Thomas Skripuletz, Matthias Schilling, Tim Hagenacker, Ernst Hund, Juliane Sachau, Ralf Baron, Helena Pernice, Sebastian Spethmann, Cyrus Khandanpour, Alexander Carpinteiro, Caroline Morbach, Katrin Hahn

**Affiliations:** 1 Amyloidosis Center, Heidelberg University Hospital, and Faculty of Medicine, Heidelberg University, Heidelberg, Germany; 2 Department of Neurology, Heidelberg University Hospital, and Faculty of Medicine, Heidelberg University, Heidelberg, Germany; 3 Department of Neurology, Medical Faculty, Neuromuscular Outpatient Clinic, RWTH Aachen University, Aachen, Germany; 4 Department of Neurology, Hannover Medical School, Hannover, Germany; 5 Amyloidosis Centre Lower Saxony, Hannover Medical School, Hannover, Germany; 6 Department of Neurology, University Hospital of Muenster, Muenster, Germany; 7 Department of Neurology, and Center for Translational Neuro- and Behavioral Sciences (C-TNBS), University Medicine Essen, Essen, Germany; 8 Division of Neurological Pain Research and Therapy, Department of Neurology, University Hospital Schleswig-Holstein, Campus Kiel, Arnold‑Heller‑Str. 3, Haus D, 24105 Kiel, Germany; 9 Amyloidosis Center Charité Berlin (ACCB), Charité University Medicine, Berlin, Germany; 10 Department of Neurology and Experimental Neurology, Charité Universitätsmedizin Berlin, corporate member of Freie Universität Berlin and Humboldt Universität zu Berlin, Campus Benjamin Franklin, Berlin, 12203 Hindenburgdamm 30 Germany; 11 Department of Cardiology, Angiology and Intensive Care Medicine, Deutsches Herzzentrum der Charité, CCM, Berlin, Germany; 12 Charité – Universitätsmedizin Berlin, corporate member of Freie Universität Berlin and Humboldt-Universität zu Berlin, Berlin, Germany; 13 Department of Internal Medicine, Oncology and Hematology, Carl von Ossietzky University Oldenburg, Oldenburg, Germany; 14 Department of Hematology and Stem Cell Transplantation, University Hospital Essen, Essen, Germany; 15 Institute of Molecular Biology, University of Duisburg-Essen, Essen, Germany; 16 West German Amyloidosis Center, University Hospital Essen, Essen, Germany; 17 Interdisciplinary Amyloidosis Center of Northern Bavaria, University Hospital Würzburg, Würzburg, Germany; 18 Dpt. Clinical Research and Epidemiology, Comprehensive Heart Failure Center (CHFC), University and University Hospital Würzburg, Würzburg, Germany; 19 Department of Internal Medicine I, Cardiology, University Hospital Würzburg, Würzburg, Germany

**Keywords:** ATTRv amyloidosis, Consensus, Germany, Polyneuropathy, Silencer, Stabilizer, Therapy, Transthyretin

## Abstract

Hereditary transthyretin (ATTRv) amyloidosis is a rare, progressive multisystem disease caused by pathogenic variants in the *transthyretin* (*TTR*) gene. ATTRv-associated Polyneuropathy (ATTRv-PN) manifests in a length-dependent sensorimotor and autonomic pattern, commonly accompanied by cardiomyopathy. In recent years, clinical trials have introduced two classes of disease modifying treatments (DMT), TTR stabilizers and gene silencers. Across populations and outcome parameters, all trials share one result disease progression was significantly slowed and patients’ span and quality of life substantially improved compared to placebo, even though a function once lost would not be retrieved. This expert consensus paper provides recommendations for the treatment of ATTRv-PN in Germany and was jointly developed by experts from the German Society of Amyloid Diseases (DGAK) and the German Neurological Society (DGN). In addition to evidence from pivotal clinical trials, the recommendations incorporate benefit assessments of the German Federal Joint Committee (G-BA). For first-line treatment, the DGAK/DGN recommends one of the two second-generation *TTR* gene-silencing agents, vutrisiran or eplontersen, for most patients with symptomatic ATTRv-PN. For patients showing even minor signs of disease progression under a TTR stabilizer, the authors recommend immediate switch to a gene silencer. Evidence is limited for or against switching between silencers or combining different agents. Informed patient participation is crucial upon initiation and adaptation of DMT. For central nervous system (CNS) involvement, none of the approved agents sufficiently crosses the blood-brain barrier. Aligned with that, there are no observed or expected CNS side effects of any of the DMTs. In the future, next-generation RNA-interference therapies, gene-editing approaches, and amyloid-depleting antibodies are expected to further expand therapeutic options.

**Supplementary Information:**

The online version contains supplementary material available at 10.1186/s42466-026-00521-4.

## Introduction

Hereditary transthyretin (ATTRv) amyloidosis with polyneuropathy (ATTRv-PN) is a rare, autosomal dominant, multisystem disorder caused by pathogenic variants in the *transthyretin* (*TTR*) gene [1, 2], leading to misfolding and aggregation of TTR and subsequent extracellular deposition of amyloid fibrils [3]. Progressive amyloid accumulation results in a length-dependent sensorimotor and autonomic neuropathy, frequently accompanied by cardiomyopathy and other organ involvement [2, 4–7]. If left untreated, ATTRv-PN is associated with substantial morbidity and premature mortality [7]. Consistently across trials, placebo-treated patients showed rapid disease progression with irreversible loss of neurological function and quality of life [8–13]. Early diagnosis and timely initiation of disease-modifying therapy are therefore critical to alter the natural history of the disease and to preserve neurological function and quality of life [6, 14–16].

In the European Union, six disease-modifying pharmacological agents are currently approved for the treatment of ATTRv-PN, depending on Coutinho disease stages (FAP stages, familial amyloid polyneuropathy) [17]. In FAP stage 1, patients are fully ambulatory, in stage 2, they use walking aids, and in stage 3, individuals are wheelchair-bound or bedridden [16, 17].

Currently available treatment options include transthyretin stabilizers (tafamidis [10], diflunisal [13]) as well as gene-silencing approaches, targeting hepatic *TTR* production (inotersen [12], patisiran [8], vutrisiran [9], eplontersen [11]). Unprecedented expansion of therapeutic options has transformed patient care and offers individualized strategies tailored to disease stage, phenotype, comorbidities, and patient preference [15, 18]. However, head-to-head comparisons of the different DMTs are lacking. As a result, clinicians must rely on indirect comparisons across pivotal trials with differing study designs, populations, endpoints, and follow-up durations. Moreover, real-world data remain limited, and long-term comparative effectiveness and safety data are still evolving [19, 20]. In this context, treatment decisions require careful consideration of the available evidence on efficacy and safety, as well as regulatory status, mode of administration, monitoring requirements, comorbid organ involvement, patient-specific factors, patient preferences, and the peculiarities of the healthcare system.

## Methods

This expert consensus provides recommendations for the specific treatment of ATTRv-PN, including initial therapy selection, longitudinal monitoring of therapeutic response, criteria for therapy modification, and considerations for discontinuation. Recommendations are based on a comprehensive review of the current literature regarding the efficacy and safety of the respective drugs, as well as on additional factors relevant to individualized treatment decisions in Germany. Particular attention is given to the methodological limitations inherent to cross-trial comparisons and to the practical implications of managing a chronic, progressive multisystem disease in specialized centers.

A structured literature search was conducted using the PubMed database, covering the period from January 1980 to March 2026. The search strategy combined the following keywords and their Boolean operators: “ATTRv amyloidosis”, “transthyretin”, “polyneuropathy”, “therapy”, “stabilizer”, and “silencer”. The search was supplemented by manual screening of reference lists of relevant articles to ensure completeness. Publications were eligible if they addressed ATTRv-PN and provided data on available disease-modifying therapies, or emerging gene-editing approaches. Priority was given to randomized controlled trials, prospective cohort studies, and high-quality observational studies. Review articles and expert opinions were considered where appropriate to contextualize evidence and clinical practice. Non-English publications and studies lacking sufficient methodological transparency were excluded. Study selection and data interpretation were guided by clinical relevance, methodological rigor, and applicability to routine care in specialized centers. Given the heterogeneity of study designs and endpoints, no formal meta-analysis was performed. Instead, evidence was synthesized narratively, with explicit consideration of cross-trial limitations and potential sources of bias. The consensus process was based on iterative expert discussions within a multidisciplinary panel. Draft recommendations were reviewed and refined in multiple rounds (Table 1).

The panel of experts was selected and appointed by the German Society of Amyloid Diseases (*Deutsche Gesellschaft für Amyloid-Krankheiten*, DGAK) and the German Neurological Society (*Deutsche Gesellschaft für Neurologie*, DGN).

## Results and recommendations

### Choice of treatment

**Table 1:** Disease-modifying therapies for ATTRv-PN approved in Germany

Therapy	Mechanism class	Mechanism of action	Route of administration	Dosing schedule	Approved PN stage	Key monitoring requirements	Pivotal study
**Tafamidis**	TTR stabilizer	Selective transthyretin tetramer stabilizer preventing dissociation into amyloidogenic monomers	Oral	20 mg once daily (PN); 61 mg once daily for ATTR-CM	Stage 1	Clinical monitoring	Coelho et al. 2012 [10]; Maurer et al. 2018 [28]
**Diflunisal**	TTR stabilizer	NSAID with transthyretin tetramer stabilizing properties	Oral	250 mg twice daily	Stage 1–2	Clinical monitoring; renal function; gastrointestinal tolerance; cardiovascular risk assessment	Berk et al. 2013 [13]
**Inotersen**	1^st^ generation *TTR* gene silencer (antisense oligonucleotide)	Antisense oligonucleotide inducing RNase-H–mediated degradation of *TTR* mRNA	Subcutaneous injection	284 mg once weekly	Stage 1–2	Clinical monitoring; platelet counts; renal function (UPCR, eGFR); vitamin A supplementation	Benson et al. 2018 (NEURO-TTR) [12]
**Patisiran**	1^st^ generation *TTR* gene silencer (RNA interference)	siRNA inducing RNA-interference–mediated degradation of *TTR* mRNA	Intravenous infusion	0.3 mg/kg every 3 weeks	Stage 1–2	Clinical monitoring; premedication required; infusion reactions; vitamin A supplementation	Adams et al. 2018 (APOLLO) [8]
**Vutrisiran**	2^nd^ generation *TTR* gene silencer (RNA interference)	GalNAc-conjugated siRNA enabling targeted hepatocyte uptake via the asialoglycoprotein receptor	Subcutaneous injection	25 mg every 3 months	Stage 1–2	Clinical monitoring; vitamin A supplementation	Adams et al. 2023 (HELIOS-A) [9]; Fontana et al. 2024 (HELIOS-B) [34]
**Eplontersen**	2^nd^ generation *TTR* gene silencer (antisense oligonucleotide)	GalNAc-conjugated antisense oligonucleotide inducing RNase-H degradation of *TTR* mRNA	Subcutaneous injection (autoinjector)	45 mg every 4 weeks	Stage 1–2	Clinical monitoring; vitamin A supplementation	Coelho et al. 2023 (NEURO-TTRansform) [11]

Overview of currently approved disease-modifying therapies for ATTRv-PN in Germany. Therapies either stabilize circulating TTR tetramers (tafamidis, diflunisal) or reduce hepatic production of TTR through antisense or RNA-interference mechanisms (inotersen, patisiran, vutrisiran, eplontersen). PN stages according to Coutinho classification: stage 1 = ambulatory without assistance; stage 2 = ambulatory with assistance; stage 3 = wheelchair-bound or bedridden. In individual cases, continuation of *TTR* gene-silencing therapy may be considered in patients progressing to stage 3 if treatment was initiated in an earlier stage and clinical benefit is still expected

A detailed review of the published evidence of efficacy and safety is provided in the supplementary materials.

#### Treatment initiation

In symptomatic patients with confirmed amyloidogenic TTR variants, treatment should be initiated as early as possible [14–16, 21]. In Germany, marketing authorizations do not require confirmation of amyloid deposits by tissue biopsy [22–27]. In asymptomatic TTR variant carriers, there is no approved treatment strategy to prevent disease onset. Variant carriers with histologically proven tissue amyloid deposition but no typical disease symptoms or findings of disease manifestation in an initial comprehensive set of diagnostic tests (yet) should be strictly monitored with individual treatment considerations provided by a multidisciplinary board of experts [28].

All six DMTs are approved for Coutinho stage 1 ATTRv-PN [22–27]. In stage 2, tafamidis is not approved, whereas the other DMTs remain approved for use in this stage [22–25, 27].

Based on published evidence [8–13, 19, 20, 29–37] on efficacy, side effects, and treatment burden (for a detailed overview, see supplementary materials), the panel recommends using one of the two second-generation RNA silencers, the small interfering RNA (siRNA) agent vutrisiran [9] or the antisense oligonucleotide (ASO) eplontersen [11], as first-line therapy. A recent meta-analysis applies for the first time a statistical model to compare the different phase 3 trials, supporting this suggestion [38]. There is no head-to-head comparison study available between vutrisiran and eplontersen, directly. Based on mechanisms of action, published trial data, and recommendations by the federal joint committee [39, 40], none of the two can be considered superior in overall efficacy. Vutrisiran has been studied and approved for ATTR-CM as well [9, 35, 36], whereas the respective trial for eplontersen is still ongoing. Regarding autonomic symptoms, eplontersen has been studied in more detail and shown to be effective [11]. Patisiran has a significantly higher treatment burden over vutrisiran, and inotersen comes with greater safety issues and monitoring requirements; they should therefore be considered second-line options. If patients are stable under patisiran and explicitly prefer DMT continuation over DMT switch, there is no medical reasoning not to abide with the patients’ preference. The authors acknowledge, however, that both long-term i.v. administration and repeated exposure to corticosteroid premedication cause a significant treatment burden that represents a relevant limitation of continuous patisiran treatment. If patients explicitly prefer oral over subcutaneous route of administration, tafamidis (20 mg or 61 mg in patients with mixed phenotype) would be the first choice. Since market authorization in 2012, trial and real-world data have shown that very early FAP1 patients with the p.(Val50Met) TTR variant profit best from this DMT, especially if female [20, 31, 41]. With responder rates ~ 70% and very minor side effects only, tafamidis (20 mg or 61 mg in patients with mixed phenotype) can be considered as first-line therapy in these specific cohorts. However, the expert panel acknowledges tafamidis to be rather a bridging DMT with immediate switch to a more efficacious silencer upon any indication of disease progression. Diflunisal, an orally administered non-steroidal anti-inflammatory drug (NSAID) with disease-modifying effects mediated by TTR stabilization might be equally efficacious as tafamidis [13] (no head-to-head comparison available) but has a more relevant safety profile (potential gastrointestinal, cardiac, and renal toxicity) [27]. Acoramidis is a TTR stabilizer with enhanced binding to both thyroxine binding sites. However, it has not been studied in ATTRv-PN and is currently approved for ATTR-CM only [42, 43].

#### Treatment switch

Patients under DMT should be regularly monitored in terms of side effects and treatment response [15, 16]. Disease progression is defined by any additional and/or worsening signs and/or symptoms that are specific to the disease. Other than in clinical trial settings, the expert panel does not recommend using one specific score or instrument to measure progression but to choose a reasonable combination of tests conducted by trained examiners who should, ideally, be identical between visits. Detailed recommendations on follow-up examinations in Germany have been previously published by the expert panel [16].

If ATTRv-PN is progressive under a stabilizer, the authors recommend immediate switch to a second-generation silencer. If neuropathy is progressive under a second-generation silencer, the panel advises to measure serum TTR (i.e., prealbumin) levels in order to control for suppression efficiency. However, there is no evidence on whether or not switch from vutrisiran to eplontersen or vice versa is beneficial for patient outcomes. There is also no direct evidence that eplontersen addresses autonomic neuropathy better than vutrisiran or that vutrisiran targets cardiomyopathy better than eplontersen. The panel acknowledges that in individual cases, disease progression justifies a switch between substance classes even though there is no supporting evidence yet.

Further treatment adaptation by combining a silencer with a stabilizer comes with significant costs, and benefits and risks remain to be systematically studied. Subgroup analyses from HELIOS-B and real-world cohorts suggest a potential signal toward improved outcomes, yet studies were not powered for definitive comparisons and cumulative costs are considerable [36, 44]. Mechanistically, TTR tetramers are more prone to dissociation at low concentrations [45]. In presence of a stabilizer, the silencer-influenced stoichiometry with relative overabundance of drug over target might actually justify attempts of combination therapy in severely progressive cases. The panel suggests that this individual decision should be supported by a multidisciplinary board of experts, and coverage of costs should be clarified with the respective health insurance provider in advance.

#### Treatment discontinuation

None of the approved DMTs has been systematically studied in FAP stage 3 patients, specifically. Learning from response profiles to tafamidis, it is conceivable that stabilizers are efficient in advanced disease stages [31]. For silencers, however, single case reports suggest that even at a non-ambulatory stage of disease, patients might actually profit [46]. For patients losing ambulation while already under DMT with a silencer, the panel recommends continuing DMT and consider some aspects discussed in the section “treatment switch” above. Discontinuation of treatment upon progression is considered ethically questionable. Despite that, the authors acknowledge that obtaining coverage authorization by insurance can be a tedious process. It is being recommended discussing individual cases within the frame of a multidisciplinary board of experts and to further study the range of effects of available DMTs in research settings.

### Systemic effects and organ manifestations

Years to decades before systemic disease onset, TTR amyloid can form at different musculoskeletal sites, which results in manifestations such as (bilateral) carpal tunnel syndrome, lumbar spinal stenosis, and ruptures of biceps or other large tendons being considered a diagnostic red flag [47–50]. Even though all can mimic symptoms of neuropathy, there are no approved DMTs that could be used to prevent the development of systemic symptoms. The panel advises that patients with TTR amyloid (according to amyloid subtyping) in a soft tissue biopsy should undergo neurological and cardiological assessments and be genetically tested for amyloidogenic *TTR* variants. If positive (ATTRv), treatment should be initiated upon minor signs or symptoms of neuropathy onset. If negative (ATTRwt), the earliest signs of cardiomyopathy (e.g. ECG, NT-proBNP, high sensitive troponin, echocardiography) should prompt to tightening the diagnosis of ATTRwt by bone scan scintigraphy and/or cardiac MRI. Importantly, AL amyloidosis should be excluded at an early stage by serum free light chain measurement and immunofixation.

Cardiac involvement (ATTR-CM) most significantly contributes to morbidity and mortality [51–53]. Interstitial amyloid deposition within the myocardium leads to restrictive cardiomyopathy characterized by ventricular wall thickening and diastolic dysfunction. Clinical manifestations usually include heart failure with preserved ejection fraction, arrhythmias (particularly atrial fibrillation), and conduction disturbances, eventually requiring pacemaker implantation. Because cardiac involvement commonly occurs also in patients presenting primarily with neuropathy, regular cardiological evaluation is recommended as part of routine follow-up [54]. For ATTR-CM, three DMTs are currently approved: tafamidis (at a dose of 61 mg instead of 20 mg) [29], acoramidis [42], and vutrisiran [35]. The efficiency of eplontersen for ATTR-CM is currently being studied in a clinical Phase III trial [55–57].

In patients with ATTRwt-CM, where PN is likely more common than previously thought [58–62], outcome parameters unfortunately do not satisfyingly cover treatment response of neuropathy to any of the above DMTs. Mechanistically, however, it is conceivable that effects will not only apply for cardiac amyloid deposition, but for the peripheral nervous system as well.

Amyloid deposition may occur in the vitreous body, retina, and other ocular structures, potentially leading to visual impairment [63, 64]. Vitreous opacities are among the most characteristic ocular findings and may require vitreoretinal surgery in advanced cases. Other reported manifestations include glaucoma, abnormal conjunctival vessels, and retinal vascular changes. Importantly, ocular amyloid deposition may continue to progress in patients receiving systemic DMT [65].

Central nervous system (CNS) involvement is likely to become a relevant clinical issue in the near future [66, 67]. Specific TTR variants have been associated with primary leptomeningeal manifestation, progressive cognitive impairment, seizures, microbleeds, atypical parenchymal or subarachnoid hemorrhages, or stroke-like episodes due to amyloid deposition in cerebral vessels and meninges [68, 69]. Among liver transplanted patients carrying the p.(Val50Met) mutation, 30% develop initial signs of CNS involvement about 15 years after transplantation [70, 71]. This observation underscores that TTR production in the choroid plexus remains unaffected when therapeutic strategies are limited to targeting hepatic synthesis.

For currently approved DMTs, the blood–brain barrier represents a major pharmacokinetic hurdle, as it can only be crossed by very small and preferably uncharged molecules. ASO and RNAi molecules are unable to penetrate, and tafamidis has been measured in CSF in very low levels compared to its blood concentration - likely insufficient to achieve meaningful stabilization of TTR within the CNS [72]. Small clinical case series, mostly focusing on patients with specific CNS-associated variants, show controversial but mostly discouraging results for CNS improvement under tafamidis [72–76]. With that in mind, two conclusions are relevant to clinical practice and treatment considerations: First, none of the currently approved DMTs is expected to cause clinically relevant CNS side effects, either through off-target effects or by suppressing TTR levels in the CSF. Second, improved survival under effective DMTs allows more time for CNS manifestations of ATTRv amyloidosis to emerge, making them an increasingly important clinical concern [66]. Given the fact that TTR stabilizers have been available in Germany since 2012 and gene-silencers since 2018, clinicians should anticipate the potential emergence of patients presenting with CNS amyloidosis independent of liver transplantation. Given its known capability to cross the blood-brain-barrier and some experimental data indicating its stabilizing effect on TTR tetramers, tolcapone might be an off-label treatment option of ATTRv-CNS amyloidosis [77], requiring multiple daily doses for as stable as possible CSF levels and consequent monitoring of liver toxicity. Symptomatic treatment might include common anti-seizure drugs. Benefit-risk considerations for oral anticoagulants in patients with high risk of cardiogenic embolisms and, at the same time, of cerebral hemorrhage, remain to be elaborated.

### Prescription and costs

All DMTs approved for ATTRv-PN are associated with substantial costs, reflecting their status as orphan drugs and the complexity of their development and manufacture. Health technology assessments in Europe, including those conducted in Germany, have evaluated cost-effectiveness based on clinical benefit, quality-adjusted life years (QALYs), and long-term disease modification [78]. However, robust comparative cost-effectiveness analyses remain limited [79, 80]. In clinical practice, economic considerations must therefore be balanced against demonstrated efficacy, safety, and the potential to prevent irreversible neurological and systemic progression.

In Germany, prescription of these DMTs is typically initiated and supervised by specialists experienced in the diagnosis and management of amyloidosis, particularly neurologists in certified amyloidosis or neuromuscular centers, preferably in interdisciplinary collaboration with cardiologists and other specialists. Given the complexity of monitoring and the need for structured longitudinal assessment, as well as the potential to participate in clinical trials for innovative therapeutic approaches, treatment should be embedded in specialized care pathways [14–16, 81].

The high costs of therapy raise important ethical considerations, including equitable access, responsible resource allocation within the statutory health insurance system, and the obligation to ensure that patients with rare diseases are not disadvantaged. In the German reimbursement framework, ATTRv-PN DMTs are recognized as *Praxisbesonderheit*, acknowledging their exceptional cost profile and protecting prescribing physicians from undue budgetary constraints. Transparent documentation of indication, disease stage, and therapeutic response is nevertheless essential to support appropriate and sustainable use.

### Other factors relevant for individual treatment decisions

Overall, both TTR stabilizing therapies and *TTR* gene silencing approaches have demonstrated clinically meaningful efficacy in slowing disease progression and improving patient-reported outcomes (PROMs) in ATTRv-PN. The increasing number of approved DMTs provides important opportunities for individualized treatment strategies tailored to disease stage, organ involvement, comorbidities, and patient preferences (see also Table 2). Administration route and treatment logistics represent additional important considerations. Tafamidis and diflunisal are administered orally, whereas patisiran requires premedication-supported intravenous infusion every three weeks and therefore access to an infusion facility. In contrast, the second-generation gene-silencing therapies vutrisiran and eplontersen are administered subcutaneously at longer intervals (every three months and once monthly, respectively), which may substantially reduce treatment burden and facilitate outpatient management. Eplontersen is available as an autoinjector that allows home-based self-administration after appropriate patient training. In certain situations, support by home-care nursing services may further facilitate treatment delivery and improve adherence. Inotersen is also administered subcutaneously once weekly and may be self-administered, but requires regular laboratory monitoring because of the risk of thrombocytopenia and renal adverse events.

**Table 2:** Practical considerations for disease-modifying therapies in ATTRv-PN in Germany, summarizing strengths, limitations, administration logistics, monitoring burden, and typical use considerations

Therapy	Key strengths	Key limitations/cautions	Administration logistics	Monitoring burden	Typical use considerations
**Tafamidis**	Oral; generally well tolerated; established long-term experience; also proven benefit in ATTR-CM	PN efficacy mainly shown in early-stage disease; may be insufficient in rapidly progressive phenotypes	Oral intake once daily; low organizational complexity	**Low** (clinical monitoring)	Not recommended if large fiber PN is present; particularly relevant when cardiac phenotype is prominent
**Acoramidis**	Oral; generally well tolerated; proven benefit in ATTR-CM with strong evidence regarding ATTRv-CM	No approval for ATTRv-PN	Oral intake twice daily; low organizational complexity	**Low** (clinical monitoring)	Based on current evidence, recommendation for ATTRv-CM, only
**Diflunisal**	Oral; disease-modifying effect demonstrated in RCT; additional stabilizer option	NSAID class risks: renal dysfunction, GI bleeding/ulceration, fluid retention, cardiovascular risk; drug–drug interactions possible	Oral intake twice daily; requires risk stratification	**Moderate** (clinical monitoring; renal function; GI/CV safety surveillance)	Not recommended if large fiber PN is present; avoid/limit use in renal disease, significant heart failure, or high GI bleeding risk
**Inotersen**	Weekly s.c. injection; robust clinical effect across genotypes; no infusion center required	Thrombocytopenia and glomerulonephritis risk; requires structured lab monitoring; adherence to monitoring is essential; vitamin A supplementation required	Weekly injections (often self-administered); practical for motivated patients with reliable follow-up	**High** (clinical monitoring; platelets; UPCR/eGFR; safety-driven schedule)	Not recommended since availability of 2^nd^ generation *TTR* gene silencers
**Patisiran**	Strong evidence base; improves neurological endpoints and QoL; supportive cardiac effects in subgroups	IV infusion and premedication required; infusion reactions; requires infusion-capable center; time burden; vitamin A supplementation required	Infusion every 3 weeks (~80 min) plus premedication; logistics may limit access	**Moderate** (clinical monitoring; infusion reaction surveillance)	Not recommended since availability of 2^nd^ generation *TTR* gene silencers
**Vutrisiran**	Q3-month s.c. injection; no infusion/premedication; favorable tolerability; strong efficacy with modern delivery platform	Long-term comparative data still evolving; vitamin A supplementation required	Quarterly s.c. injection; can be administered outside infusion centers	**Low** (clinical monitoring)	Often preferred for many stage 1–2 PN patients because of convenience and low logistical burden; continuation after progression to stage 3 may be considered if started earlier (individualized)
**Eplontersen**	Monthly s.c. injection; autoinjector for home use; substantial TTR lowering and clinical benefit; favorable safety profile vs. inotersen	Long-term real-world comparative data still emerging; vitamin A supplementation required	Monthly s.c. injection (often self-administered after training)	**Low** (clinical monitoring)	Consider as a convenient, home-based gene-silencing option for stage 1–2 PN; continuation after progression to stage 3 may be considered if started earlier (individualized)

Selection should incorporate disease stage, rate of progression, organ involvement (notably cardiac and renal), feasibility of structured monitoring, and patient preferences. Evidence is largely derived from pivotal trials rather than direct head-to-head comparisons. In clinical practice, selection of disease-modifying therapy for ATTRv-PN should be individualized and ideally performed in specialized amyloidosis centers. Factors influencing treatment choice include disease stage, rate of neurological progression, presence and severity of cardiac or renal involvement, expected treatment adherence, and the feasibility of structured safety monitoring. Patient preferences and logistical considerations – such as the ability to attend infusion visits or perform self-injections – also play an important role. Given the absence of direct head-to-head randomized comparisons between currently approved therapies, treatment decisions should rely on available efficacy and safety data from pivotal trials together with real-world experience and multidisciplinary evaluation

Within the framework of the early benefit assessment according to the German Pharmaceutical Market Restructuring Act (*Arzneimittelmarktneuordnungsgesetz*, AMNOG), the Federal Joint Committee (*Gemeinsamer Bundesausschuss*, G-BA) evaluates the additional benefit of newly approved therapies compared with standard care [39, 40]. For patients with ATTRv-PN, patisiran was assessed as providing a considerable additional benefit based on the results of the APOLLO trial. In contrast, the additional benefit of inotersen was classified as not quantifiable, reflecting uncertainties regarding the magnitude of benefit despite statistically significant improvements in clinical outcomes. For vutrisiran, the G-BA determined a minor additional benefit compared with patisiran, based on advantages in treatment administration and overall treatment burden while maintaining comparable efficacy [40]. For eplontersen, the Institute for Quality and Efficiency in Health Care (*Institut für Qualität und Wirtschaftlichkeit*, IQWIG) concluded that there are no suitable data comparing eplontersen and the comparator therapy vutrisiran defined by the GB-A [39]. Such assessments may influence reimbursement conditions and prescribing patterns but do not replace individualized clinical decision-making in specialized amyloidosis centers.

All gene-silencing therapies reduce circulating TTR levels and therefore require vitamin A supplementation, as the resulting decline in TTR leads to lower serum retinol levels. In Germany, vitamin A supplementation is generally not reimbursed by statutory health insurance and therefore represents an additional out-of-pocket expense for patients. This aspect should be discussed during treatment counseling.

Patients having undergone orthotopic liver transplantation (OLT) prior to pharmacological DMT availability, were systematically excluded from pivotal phase 3 trials. Driven by ongoing deposition of wild-type TTR onto pre-existing amyloid fibrils [82], it has commonly been observed now that these individuals experience significant disease progression and require additional treatment. In a dedicated phase 3b trial, patisiran demonstrated sustained TTR suppression and clinical stabilization without evidence of allograft rejection or interaction with immunosuppressive therapy [33]. Retrospective data suggest that inotersen may stabilize neuropathy in selected post-transplant patients, albeit with safety concerns [83], while evidence for tafamidis remains limited to small observational series [84]. In progressive post-OLT disease, treatment with a TTR gene silencer or stabilizer appears justified with careful hepatic monitoring.

### Future perspectives

Novel disease-modifying strategies may further reshape the therapeutic landscape. In vivo CRISPR-Cas9 gene editing targeting hepatic *TTR* production has demonstrated profound and durable TTR knockdown in early-phase studies, raising the prospect of one-time therapeutic interventions [85]. In parallel, amyloid-depleting monoclonal antibodies designed to remove deposited TTR fibrils are under active investigation, currently primarily in ATTR-CM populations. Early-phase studies with fibril-targeting antibodies such as PRX004 and NI006 have provided proof-of-concept for cardiac amyloid reduction, and ongoing placebo-controlled phase 3 programs aim to confirm clinical benefit [86].

The TRITON-PN and TRITON-CM programs evaluating the third-generation RNAi *TTR* gene-silencer nucresiran will further clarify long-term efficacy and safety across neurological and cardiac phenotypes.

**Fig. 1:**
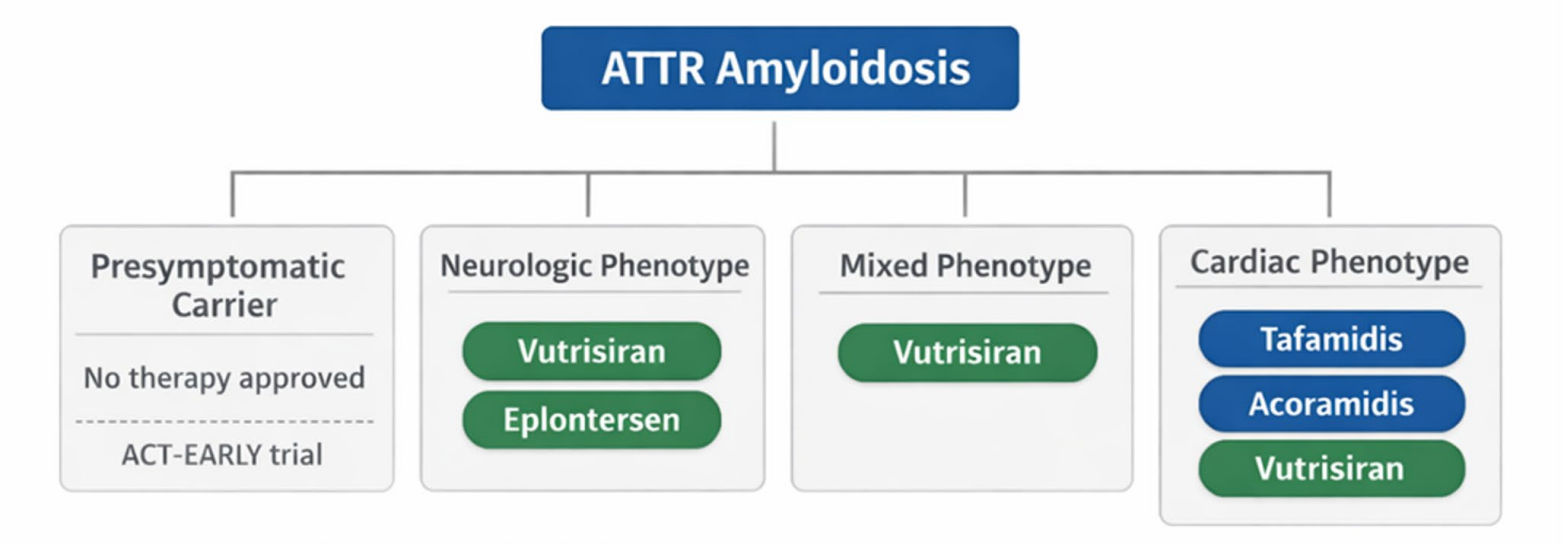
Therapeutic algorithm for ATTR amyloidosis according to clinical phenotype. Schematic overview of treatment strategies for ATTRv amyloidosis based on the predominant clinical phenotype. In presymptomatic TTR variant carriers, no disease-modifying therapy is currently approved; participation in the placebo-controlled ACT-EARLY study may be considered. For patients with a predominantly neurologic phenotype (ATTRv-PN), the second-generation *TTR* gene-silencing agents vutrisiran and eplontersen represent the preferred therapeutic options. In Germany, these agents are approved for ATTRv-PN stages 1 and 2; continuation of therapy may be considered in selected patients who progress to stage 3, provided treatment was initiated earlier. In mixed neurologic-cardiac phenotypes, vutrisiran represents an important treatment option due to its efficacy in both neurologic and cardiac manifestations. For patients with a predominantly cardiac phenotype (ATTR-CM, both wild-type or variant), disease-modifying therapy includes tafamidis, acoramidis, or vutrisiran

Finally, presymptomatic intervention is gaining momentum. The ACT-EARLY phase 3 randomized, placebo-controlled trial investigates prophylactic use of acoramidis in asymptomatic TTR variant carriers, reflecting recognition that amyloid deposition precedes overt disease and that penetrance varies by genotype and geographic background [4] (Figure 1).

### Summary

The therapeutic landscape of ATTRv-PN has evolved substantially with the introduction of disease-modifying therapies targeting different steps of the amyloid cascade. Currently available options include TTR stabilizers (tafamidis, diflunisal) and *TTR* gene-silencing therapies that suppress hepatic transthyretin production (inotersen, patisiran, vutrisiran, and eplontersen).

These recommendations represent expert consensus guidance for treatment of ATTRv-PN in Germany, developed jointly by experts from the DGAK and the DGN. In addition to evidence from pivotal clinical trials, the recommendations incorporate benefit assessments of the G-BA within the AMNOG framework.

Based on the available evidence and current clinical practice in Germany, the DGAK/DGN consensus recommends first-line therapy with one of the two second-generation *TTR* gene-silencing agents, vutrisiran or eplontersen, for most patients with symptomatic ATTRv-PN (Figure 1). These agents provide profound suppression of circulating TTR, favorable safety profiles, and convenient subcutaneous administration.

At the same time, the therapeutic landscape of ATTR amyloidosis continues to evolve, with emerging approaches including next-generation RNAi therapies, in vivo gene-editing strategies targeting *TTR* production, and amyloid-depleting antibodies currently investigated mainly in ATTR-CM, as well as studies exploring presymptomatic treatment of TTR variant carriers (Figure 1).

## Supplementary Information

Supplementary Material 1

## Data Availability

No datasets were generated or analysed during the current study.

## Abbreviations

AMNOG: Arzneimittelmarktneuordnungsgesetz (German Pharmaceutical Market Restructuring Act)
ASO: Antisense oligonucleotide
ATTR-CM: Transthyretin amyloidosis with cardiomyopathy
ATTR-PN: Transthyretin amyloidosis with polyneuropathy
ATTRv amyloidosis: Hereditary transthyretin amyloidosis
COMPASS-31: Composite Autonomic Symptom Score 31
CNS: Central nervous system
CTS: Carpal tunnel syndrome
DGAK: Deutsche Gesellschaft für Amyloid-Krankheiten (German Society of Amyloid Diseases)
DGN: Deutsche Gesellschaft für Neurologie (German Neurological Society)
DMT: Disease-modifying treatments
EAN: European Academy of Neurology
GalNAc: N-acetylgalactosamine
G-BA: Gemeinsamer Bundesausschuss (German Federal Joint Committee)
HRV: Heart rate variability
ISA: International Society of Amyloidosis
mBMI: Modified body mass index
mNIS + 7: Modified Neuropathy Impairment Score + 7
mRNA: Messenger ribonucleic acid
NCS: Nerve conduction studies
NfL: Neurofilament light chain
NIS: Neuropathy Impairment Score
NIS + 7: Neuropathy Impairment Score + 7
NIS-LL: Lower-limb Neuropathy Impairment Score
OLT: Orthotopic liver transplantation
PND: Polyneuropathy Disability
PNS: Peripheral Nerve Society
QALYs: Quality-adjusted life years
QST: Quantitative sensory testing
RISC: Ribonucleic acid-induced silencing complex
RNAi: Ribonucleic acid interference
R-ODS: Rasch-built Overall Disability Scale
siRNA: Small interfering ribonucleic acid
6-MWT: 6-Minute Walk Test
TTR: Transthyretin
