# Supplementary material for: Disease modifying treatment of hereditary transthyretin amyloidosis with polyneuropathy in Germany – expert consensus of the German society of amyloid diseases (DGAK) and the German neurological society (DGN)
Source: Neurol Res Pract. 2026 Aug 11;8(1):63. doi: 10.1186/s42466-026-00521-4 (PMC13466188; doi:10.1186/s42466-026-00521-4)
Supplement: Supplementary file 1 [file 42466_2026_521_MOESM1_ESM.docx]

**Disease Modifying Treatment of hereditary transthyretin amyloidosis with polyneuropathy in Germany –**

**Expert Consensus of the German Society of Amyloid Diseases (DGAK) and the German Neurological Society (DGN)**

Markus Weiler^1,2^*, Maike F. Dohrn^3^*, Stefan Gingele^4,5^, Thomas Skripuletz^4,5^, Matthias Schilling^6^, Tim Hagenacker^7^, Ernst Hund^1,2^, Juliane Sachau^8^, Ralf Baron^8^, Helena Pernice^9,10^, Sebastian Spethmann^9,11,12^, Cyrus Khandanpour^13^, Alexander Carpinteiro^14,15,16^, Caroline Morbach^17,18,19^, Katrin Hahn^9,10^

**Supplementary material: review of published evidence on all approved disease-modifying treatments (DMTs)**

***Treatment options***

Consistent with observations from natural history studies, the placebo groups of recently completed pivotal therapeutic trials in ATTRv-PN demonstrated rapid neurological deterioration (1-4). This worsening was accompanied by a parallel decline in health-related quality of life, highlighting the progressive and disabling nature of untreated disease and emphasizing the importance of early therapeutic intervention. A primary objective of treatment is to halt or substantially slow disease progression. If disease-modifying therapies cannot be used because of advanced neurological disability (polyneuropathy stage 3) or due to contraindications related to relevant comorbidities, management should focus on interdisciplinary symptomatic treatment. Given the multisystemic nature of ATTRv amyloidosis, symptomatic management must be individualized according to the clinical phenotype and organ involvement. Therapeutic measures may include multimodal pain management, surgical decompression for carpal tunnel syndrome, pacemaker implantation in cardiac conduction disease, heart transplantation in advanced cardiomyopathy, dialysis or kidney transplantation in renal involvement, and vitreoretinal surgery for ocular manifestations. Optimal care therefore requires close interdisciplinary collaboration between neurology, cardiology, nephrology, ophthalmology, and other specialties. (5, 6)

***Eligibility for disease-modifying therapy***

In Germany, the current prerequisites for initiating disease-modifying pharmacotherapy include genetic confirmation of a pathogenic, i.e. amyloidogenic variant in the *TTR* gene and the presence of symptomatic neuropathy with preserved ambulation (polyneuropathy stage 1 or 2) (7-12). These criteria reflect the inclusion criteria of the pivotal clinical trials that established the efficacy of currently approved therapies (1-4, 13, 14). For asymptomatic TTR variant carriers, disease-modifying therapy is currently not approved even when amyloid deposits are detected in biopsy specimens. Nevertheless, in specific circumstances, for example when amyloid deposits are identified in tissue obtained during carpal tunnel surgery, individualized decisions regarding early treatment initiation may be discussed within interdisciplinary amyloidosis boards (15). Continuation of *TTR* gene-silencing therapies may also be considered in selected patients who progress to polyneuropathy stage 3 during the course of treatment, provided that therapy had been initiated in an earlier disease stage (16). In such cases, treatment decisions should be made individually in specialized amyloidosis centers after multidisciplinary evaluation and careful assessment of the expected clinical benefit. At present, several pharmacological agents are approved in Germany for disease-modifying treatment of ATTRv amyloidosis. Orthotopic liver transplantation (OLT), which was introduced in the early 1990s as the first causal therapy by replacing the primary source of variant TTR production, is nowadays rarely performed because modern pharmacological therapies can suppress hepatic *TTR* synthesis or stabilize circulating TTR with substantially lower procedural risks (17). An overview of currently available disease-modifying therapies is provided in Table 1.

***Transthyretin-stabilizing therapies***

*Tafamidis*
Tafamidis meglumine is an orally administered benzoxazole derivative that selectively binds circulating TTR and stabilizes the tetrameric structure of the protein. By preventing tetramer dissociation – the rate-limiting step in amyloid formation – tafamidis reduces the generation of misfolded monomers capable of forming amyloid fibrils (18). Tafamidis 20 mg/day has been approved in Europe since 2011 for patients with ATTRv-PN stage 1 (11). In the pivotal randomized controlled trial, tafamidis treatment resulted in clinical improvement in the lower-limb Neuropathy Impairment Score (NIS-LL) in approximately 60% of patients compared with 38% in the placebo group after 18 months (1). Subsequent studies confirmed stabilization of quality of life and modified body mass index (mBMI), and long-term follow-up data indicate that earlier initiation of therapy is associated with better preservation of neurological function (18-20). In addition to its use in neuropathy, tafamidis has demonstrated clinical efficacy in ATTR-CM. In a large phase 3 trial including 441 patients, tafamidis significantly improved survival, reduced cardiovascular hospitalizations, and improved functional capacity compared with placebo (21).

*Diflunisal*
Diflunisal is a non-steroidal anti-inflammatory drug that stabilizes the TTR tetramer and thereby inhibits dissociation into amyloidogenic monomers. In a randomized controlled trial including 130 patients with ATTRv-PN, treatment with diflunisal significantly slowed neurological disease progression compared with placebo, as reflected by a smaller increase in the Neuropathy Impairment Score +7 (NIS+7) (4). Based on these data, diflunisal has recently been approved in Germany for the treatment of ATTRv-PN in patients with stage 1 or stage 2 disease (12). The regulatory decision was primarily supported by the randomized controlled trial, which demonstrated a clinically meaningful delay in neurological deterioration in treated patients (4). Diflunisal is administered orally and represents an additional disease-modifying therapeutic option for patients with early-stage ATTRv-PN. However, careful patient selection and monitoring are required because of potential adverse effects including renal impairment, gastrointestinal toxicity, and cardiovascular complications (12).

*Acoramidis*
Acoramidis is a next-generation TTR stabilizer designed to mimic the protective TTR variant p.Thr139Met (Thr119Met) (22) and to achieve near-complete stabilization of the TTR tetramer. In contrast to tafamidis and diflunisal, acoramidis is currently approved for the treatment of ATTR-CM, but not for ATTRv-PN. In the randomized phase 3 ATTRibute-CM trial, treatment with acoramidis significantly improved survival, reduced cardiovascular hospitalizations, and improved functional capacity and quality-of-life measures compared with placebo in patients with ATTR-CM (23, 24). These results confirm the therapeutic potential of highly potent TTR stabilization strategies in cardiac ATTR amyloidosis. Although acoramidis has not been studied in randomized trials specifically targeting ATTRv-PN, it represents an additional disease-modifying option for patients with predominant cardiac involvement.

***Gene-silencing therapies targeting hepatic* TTR *production***

A major therapeutic advance in ATTRv amyloidosis has been the development of gene-silencing therapies that reduce hepatic production of TTR. These therapies act by degrading *TTR* messenger ribonucleic acid (mRNA) or preventing its translation, thereby reducing synthesis of both mutant and wild-type TTR. Because TTR is produced predominantly in hepatocytes, effective therapy requires efficient delivery of the therapeutic molecule to liver cells (25, 26). Since 2018, two gene-silencing therapies have been available in Germany: the antisense oligonucleotide (ASO) Inotersen (3) and the RNA interference (RNAi) therapeutic patisiran (2). Subsequently, second-generation RNAi and antisense technologies have led to the development of vutrisiran (13) and eplontersen (14), which provide improved pharmacokinetics and simplified dosing schedules.

*Inotersen*
Inotersen (7) is a single-stranded ASO that reduces TTR production by binding complementary *TTR* pre-mRNA and inducing RNase-H-mediated degradation of the transcript. The phase 3 NEURO-TTR trial enrolled 172 patients with ATTRv-PN stages 1–2 and demonstrated that weekly subcutaneous administration significantly slowed neurological disease progression compared with placebo. Patients receiving inotersen showed significant improvements in the modified Neuropathy Impairment Score +7 (mNIS+7) and in the Norfolk Quality-of-Life Diabetic Neuropathy questionnaire after 66 weeks of treatment (3). Treatment resulted in a substantial reduction of circulating TTR serum concentrations, typically to approximately 25% of baseline levels. Clinical benefits were observed across TTR variant types and disease stages and were independent of concomitant cardiac involvement. The most relevant safety concern is thrombocytopenia, which requires regular platelet monitoring during therapy. Additional adverse events include nausea, fever, and rare cases of glomerulonephritis. Because TTR reduction leads to decreased circulating retinol levels, vitamin A supplementation is recommended (3, 7).

*Patisiran*
Patisiran (8) is a double-stranded small interfering RNA (siRNA) therapeutic that selectively degrades *TTR* mRNA through the endogenous RNAi pathway (27). The siRNA is encapsulated in lipid nanoparticles that protect the molecule from degradation and facilitate delivery into hepatocytes. Following intracellular release, the siRNA is incorporated into the RNA-induced silencing complex (RISC), which mediates sequence-specific cleavage of *TTR* mRNA (28). The randomized APOLLO phase 3 trial demonstrated that patisiran significantly improved neuropathy impairment, autonomic symptoms, and health-related quality of life compared with placebo. Treatment resulted in an approximately 80% reduction in serum TTR levels and led to clinically meaningful improvements in neurological function (2, 29, 30). Cardiac subgroup analyses demonstrated improvements in left ventricular wall thickness, global longitudinal strain, and NT-proBNP concentrations, suggesting beneficial effects in patients with ATTRv-CM (31). Patisiran is administered as an intravenous infusion every three weeks and requires premedication with dexamethasone, paracetamol, and combined H1/H2 receptor blockade to mitigate the risk of infusion-related reactions. Peripheral edema and infusion reactions represent the most frequently reported adverse events. Vitamin A supplementation is required (8).

*Vutrisiran*
Vutrisiran (9) is a second-generation siRNA therapeutic in which the siRNA molecule is conjugated to a triantennary N-acetylgalactosamine (GalNAc) ligand enabling targeted uptake into hepatocytes via the asialoglycoprotein receptor (32). This delivery strategy allows subcutaneous administration and prolongs the duration of action, permitting dosing only once every three months. The HELIOS-A phase 3 trial demonstrated that vutrisiran significantly improved neuropathy impairment scores, quality-of-life measures, and functional outcomes compared with an external placebo cohort derived from the APOLLO trial. Serum TTR reductions were comparable to those observed with patisiran (13). Vutrisiran demonstrated a favorable safety profile. The most frequently reported adverse events included arthralgia, dyspnea, and decreased retinol levels. As with other TTR-lowering therapies, vitamin A supplementation is recommended (9). The subsequent HELIOS-B trial further demonstrated clinical benefits of vutrisiran in ATTR-CM, including reductions in mortality and cardiovascular events while preserving functional capacity and quality of life (33, 34).

*Eplontersen*
Eplontersen (10) is a second-generation ASO designed to enhance hepatocyte uptake through conjugation with a triantennary GalNAc ligand. Like inotersen, eplontersen reduces TTR production by inducing RNase-H-mediated degradation of *TTR* mRNA, but the GalNAc conjugation improves hepatic targeting and allows lower doses with less frequent administration (14). Eplontersen is administered as a monthly subcutaneous injection and is available in an autoinjector device for self-administration following patient training (10). The NEURO-TTRansform phase 3 trial demonstrated that eplontersen significantly reduced serum TTR levels and improved neurological impairment and quality-of-life outcomes compared with a historical placebo cohort derived from the NEURO-TTR trial (14). The safety profile was favorable, with lower rates of thrombocytopenia and renal complications compared with earlier antisense therapies. An additional analysis showed that patients switching from inotersen to eplontersen maintained clinical stability while demonstrating improved tolerability (35). The CARDIO-TTR trial, assessing safety and efficacy of eplontersen of TTR-associated cardiomyopathy, is still ongoing.

**Supplementary references**

1.	Coelho T, Maia LF, Martins da Silva A, Waddington Cruz M, Planté-Bordeneuve V, Lozeron P, et al. Tafamidis for transthyretin familial amyloid polyneuropathy: a randomized, controlled trial. Neurology. 2012;79(8):785-92.

2.	Adams D, Gonzalez-Duarte A, O’Riordan WD, Yang C-C, Ueda M, Kristen AV, et al. Patisiran, an RNAi therapeutic, for hereditary transthyretin amyloidosis. New england journal of medicine. 2018;379(1):11-21.

3.	Benson MD, Waddington-Cruz M, Berk JL, Polydefkis M, Dyck PJ, Wang AK, et al. Inotersen treatment for patients with hereditary transthyretin amyloidosis. New England Journal of Medicine. 2018;379(1):22-31.

4.	Berk JL, Suhr OB, Obici L, Sekijima Y, Zeldenrust SR, Yamashita T, et al. Repurposing diflunisal for familial amyloid polyneuropathy: a randomized clinical trial. Jama. 2013;310(24).

5.	Adams D, Algalarrondo V, Polydefkis M, Sarswat N, Slama MS, Nativi-Nicolau J. Expert opinion on monitoring symptomatic hereditary transthyretin-mediated amyloidosis and assessment of disease progression. Orphanet journal of rare diseases. 2021;16(1):411.

6.	Ando Y, Adams D, Benson MD, Berk JL, Planté-Bordeneuve V, Coelho T, et al. Guidelines and new directions in the therapy and monitoring of ATTRv amyloidosis. Amyloid. 2022;29(3):143-55.

7.	Akcea Therapeutics/Ionis G. Fachinformation Tegsedi® (inotersen). 2018.

8.	Alnylam Pharmaceuticals G. Fachinformation Onpattro® (patisiran). 2018.

9.	Alnylam Pharmaceuticals G. Fachinformation Amvuttra® (vutrisiran). 2018.

10.	Germany AI. Fachinformation Wainzua® (eplontersen). 2025.

11.	Pfizer Pharmaceuticals G. Fachinformation Vyndaqel® (tafamidis). 2012.

12.	Purpose Pharma G. Fachinformation Attrogy® (diflunisal). 2025.

13.	Adams D, Tournev IL, Taylor MS, Coelho T, Planté-Bordeneuve V, Berk JL, et al. Efficacy and safety of vutrisiran for patients with hereditary transthyretin-mediated amyloidosis with polyneuropathy: a randomized clinical trial. Amyloid. 2023;30(1):18-26.

14.	Coelho T, Marques Jr W, Dasgupta NR, Chao C-C, Parman Y, França Jr MC, et al. Eplontersen for hereditary transthyretin amyloidosis with polyneuropathy. Jama. 2023;330(15):1448-58.

15.	Dohrn MF, Auer-Grumbach M, Baron R, Birklein F, Escolano-Lozano F, Geber C, et al. Chance or challenge, spoilt for choice? New recommendations on diagnostic and therapeutic considerations in hereditary transthyretin amyloidosis with polyneuropathy: the German/Austrian position and review of the literature. Journal of Neurology. 2021;268(10):3610-25.

16.	Müschen LH, Körner G, Gingele S, Hänselmann A, Bavendiek U, Skripuletz T. Treatment with patisiran of a patient with hereditary transthyretin‐mediated amyloidosis with stage 3 polyneuropathy. Muscle & Nerve. 2021;64(3):E11-E3.

17.	Yamamoto S, Wilczek H, Nowak G, Larsson M, Oksanen A, Iwata T, et al. Liver transplantation for familial amyloidotic polyneuropathy (FAP): a single‐center experience over 16 years. American Journal of Transplantation. 2007;7(11):2597-604.

18.	Bulawa CE, Connelly S, DeVit M, Wang L, Weigel C, Fleming JA, et al. Tafamidis, a potent and selective transthyretin kinetic stabilizer that inhibits the amyloid cascade. Proceedings of the National Academy of Sciences. 2012;109(24):9629-34.

19.	Merlini G, Planté-Bordeneuve V, Judge DP, Schmidt H, Obici L, Perlini S, et al. Effects of tafamidis on transthyretin stabilization and clinical outcomes in patients with non-Val30Met transthyretin amyloidosis. Journal of cardiovascular translational research. 2013;6(6):1011-20.

20.	Planté-Bordeneuve V, Gorram F, Salhi H, Nordine T, Ayache SS, Le Corvoisier P, et al. Long-term treatment of transthyretin familial amyloid polyneuropathy with tafamidis: a clinical and neurophysiological study. Journal of neurology. 2017;264(2):268-76.

21.	Maurer MS, Schwartz JH, Gundapaneni B, Elliott PM, Merlini G, Waddington-Cruz M, et al. Tafamidis treatment for patients with transthyretin amyloid cardiomyopathy. New England Journal of Medicine. 2018;379(11):1007-16.

22.	Almeida M, Alves I, Terazaki H, Ando Y, Saraiva M. Comparative studies of two transthyretin variants with protective effects on familial amyloidotic polyneuropathy: TTR R104H and TTR T119M. Biochemical and biophysical research communications. 2000;270(3):1024-8.

23.	Gillmore JD, Judge DP, Cappelli F, Fontana M, Garcia-Pavia P, Gibbs S, et al. Efficacy and safety of acoramidis in transthyretin amyloid cardiomyopathy. New England Journal of Medicine. 2024;390(2):132-42.

24.	Judge DP, Gillmore JD, Alexander KM, Ambardekar AV, Cappelli F, Fontana M, et al. Long-term efficacy and safety of acoramidis in ATTR-CM: initial report from the open-label extension of the ATTRibute-CM trial. Circulation. 2025;151(9):601-11.

25.	Adams D, Hawkins PN, Polydefkis M. Oligonucleotide drugs for transthyretin amyloidosis. New England Journal of Medicine. 2018;379(21):2086-.

26.	Ioannou A, Fontana M, Gillmore JD. RNA Targeting and Gene Editing Strategies for Transthyretin Amyloidosis: A. Ioannou et al. BioDrugs. 2023;37(2):127-42.

27.	Coelho T, Adams D, Silva A, Lozeron P, Hawkins PN, Mant T, et al. Safety and efficacy of RNAi therapy for transthyretin amyloidosis. New England Journal of Medicine. 2013;369(9):819-29.

28.	Zhao Y, Huang L. Lipid nanoparticles for gene delivery. Advances in genetics. 2014;88:13-36.

29.	Adams D, Polydefkis M, González-Duarte A, Wixner J, Kristen AV, Schmidt HH, et al. Long-term safety and efficacy of patisiran for hereditary transthyretin-mediated amyloidosis with polyneuropathy: 12-month results of an open-label extension study. The Lancet Neurology. 2021;20(1):49-59.

30.	Obici L, Berk JL, González-Duarte A, Coelho T, Gillmore J, Schmidt HH-J, et al. Quality of life outcomes in APOLLO, the phase 3 trial of the RNAi therapeutic patisiran in patients with hereditary transthyretin-mediated amyloidosis. Amyloid. 2020;27(3):153-62.

31.	Solomon SD, Adams D, Kristen A, Grogan M, González-Duarte A, Maurer MS, et al. Effects of patisiran, an RNA interference therapeutic, on cardiac parameters in patients with hereditary transthyretin-mediated amyloidosis: analysis of the APOLLO study. Circulation. 2019;139(4):431-43.

32.	Springer AD, Dowdy SF. GalNAc-siRNA conjugates: leading the way for delivery of RNAi therapeutics. Nucleic acid therapeutics. 2018;28(3):109-18.

33.	Fontana M, Berk JL, Gillmore JD, Witteles RM, Grogan M, Drachman B, et al. Vutrisiran in patients with transthyretin amyloidosis with cardiomyopathy. New England Journal of Medicine. 2025;392(1):33-44.

34.	Witteles RM, Garcia-Pavia P, Damy T, Grogan M, Sheikh FH, Morbach C, et al. Vutrisiran improves survival and reduces cardiovascular events in ATTR amyloid cardiomyopathy: HELIOS-B. Journal of the American College of Cardiology. 2025;85(20):1959-70.

35.	Conceição I, Berk JL, Weiler M, Kowacs PA, Dasgupta NR, Khella S, et al. Switching from inotersen to eplontersen in patients with hereditary transthyretin-mediated amyloidosis with polyneuropathy: analysis from NEURO-TTRansform. Journal of Neurology. 2024;271(10):6655-66.
